# Continuous shear wave measurements for dynamic cardiac stiffness evaluation in pigs

**DOI:** 10.1038/s41598-023-44588-4

**Published:** 2023-10-17

**Authors:** Annette Caenen, Lana Keijzer, Stéphanie Bézy, Jürgen Duchenne, Marta Orlowska, Antonius F. W. Van Der Steen, Nico De Jong, Johan G. Bosch, Jens-Uwe Voigt, Jan D’hooge, Hendrik J. Vos

**Affiliations:** 1 Department of Cardiology, Erasmus MC University Medical Center, Rotterdam, The Netherlands; 2 Cardiovascular Imaging and Dynamics Lab, KU Leuven, Leuven, Belgium; 3 Institute for Biomedical Engineering and Technology, Ghent University, Ghent, Belgium; 4 Department of Imaging Physics, Delft University of Technology, Delft, The Netherlands; 5 Cardiology, KU Leuven, Leuven, Belgium

**Keywords:** Preclinical research, Biomedical engineering, Echocardiography

## Abstract

Ultrasound-based shear wave elastography is a promising technique to non-invasively assess the dynamic stiffness variations of the heart. The technique is based on tracking the propagation of acoustically induced shear waves in the myocardium of which the propagation speed is linked to tissue stiffness. This measurement is repeated multiple times across the cardiac cycle to assess the natural variations in wave propagation speed. The interpretation of these measurements remains however complex, as factors such as loading and contractility affect wave propagation. We therefore applied transthoracic shear wave elastography in 13 pigs to investigate the dependencies of wave speed on pressure–volume derived indices of loading, myocardial stiffness, and contractility, while altering loading and inducing myocardial ischemia/reperfusion injury. Our results show that diastolic wave speed correlates to a pressure–volume derived index of operational myocardial stiffness (R = 0.75, *p* < 0.001), suggesting that both loading and intrinsic properties can affect diastolic wave speed. Additionally, the wave speed ratio, i.e. the ratio of systolic and diastolic speed, correlates to a pressure–volume derived index of contractility, i.e. preload-recruitable stroke work (R = 0.67, *p* < 0.001). Measuring wave speed ratio might thus provide a non-invasive index of contractility during ischemia/reperfusion injury.

## Introduction

Myocardial stiffness changes dynamically throughout the cardiac cycle and is closely intertwined with the electromechanics and hemodynamics of the heart. During diastole, the heart relaxes and myocardial stiffness is low to accommodate ventricular filling, whereas during systole the heart contracts, increasing myocardial stiffness, to generate the pressure build up before ejection. It has been shown that characterization of the time-varying myocardial elastance using pressure–volume measurements can provide insights into cardiac function^1^. This is essential for increasing our understanding of cardiovascular pathophysiology and improving treatment of multiple cardiac disorders.

Ultrasound-based shear wave elastography (SWE) has been proposed as non-invasive method for measuring the dynamics of myocardial stiffness^2^. The fundamental principle of this technology is to image the propagation of acoustically induced vibrations along the cardiac wall, hence representing the propagation of a mechanical wave. The propagation speed of this wave is related to the intrinsic tissue stiffness, and higher speeds are measured in stiffer tissues^3^. By repeating a SWE measurement multiple times throughout the cardiac cycle, it is possible to construct a time-varying wave speed curve of which its characteristics are related to the myocardial stiffness dynamics^2, 4^. Indeed, the magnitude of the wave speed in diastole reflects passive myocardial stiffness: diastolic wave speed correlates to other stiffness parameters derived from the end-diastolic pressure–volume relationship^5^ and the end-diastolic stress–strain relationship^6, 7^. The magnitude of the wave speed in systole has been put forward as a measure for contractility, as it correlated strongly with end-systolic pressure during beta-adrenergic stimulation^2^. Next to the magnitude of the wave speed, the dynamics of the wave speed curve also offered insights into the isovolumic relaxation time constant^5^.

It should be noted that all previously mentioned studies measured dynamic stiffness variations in an ex vivo or open-chest animal setting to bypass the limited SWE image quality of transthoracic recordings. To investigate whether previous findings hold in an intact heart with realistic loading conditions, it is essential to perform closed-chest SWE measurements in combination with pressure–volume (PV) recordings. These measurements can also sort out the remaining uncertainties in literature how other factors such as loading but also a combination of contractility and intrinsic stiffness (in for example an infarct) affect SWE measurements in diastole and systole. A preload independence of diastolic and systolic wave speed measurements has been suggested^2^, whereas other studies showed that an increase in ventricular or intra-myocardial pressure affected wave propagation speed in a Langendorff-prepared set-up^8, 9^. We therefore hypothesized that increased ventricular pressure can induce an apparent stiffening of the myocardium due to the intrinsic material non-linearity. There is also inconclusiveness on the use of systolic wave speed as measure for contractility during ischemia/reperfusion (I/R) injury, as both a decrease^4, 6^ and increase in systolic wave speed^7^ have been reported. To elucidate the aforementioned uncertainties, the objective of this work was to investigate the relation between diastolic and systolic wave speeds and indices of loading, myocardial stiffness, and contractility derived from PV loops. We therefore applied transthoracic SWE in a pig model where we altered loading of the heart and induced myocardial I/R injury.

## Materials and methods

### Animal model

All animal procedures were approved by the local Ethical Committee for Animal Experimentation (ECD) of UZ Leuven, and were performed according to the ethical standards formulated by the European Commission (2016/63/EU). The procedures were written up following the ARRIVE guidelines. Thirteen Yorkshire-Landrace pigs (31.2 ± 4.1 kg; 6 female) in supine position were anesthetized with an intramuscular injection of tiletamine/zolazepam (4 mg/kg) and xylazine (2.5 mg/kg). Anesthesia was maintained by intravenous infusion of propofol (0.17 mg/kg/h) and remifentanil (0.3 mg/kg/min), while pigs were mechanically ventilated. Vital signs, including heart rate, oxygen saturation, and arterial blood pressure, were monitored. It should be noted that the animals were the same as in our previous study^10^, which focused on wave speed measurements after mitral valve closure.

### Experimental protocol

**Figure 1:**
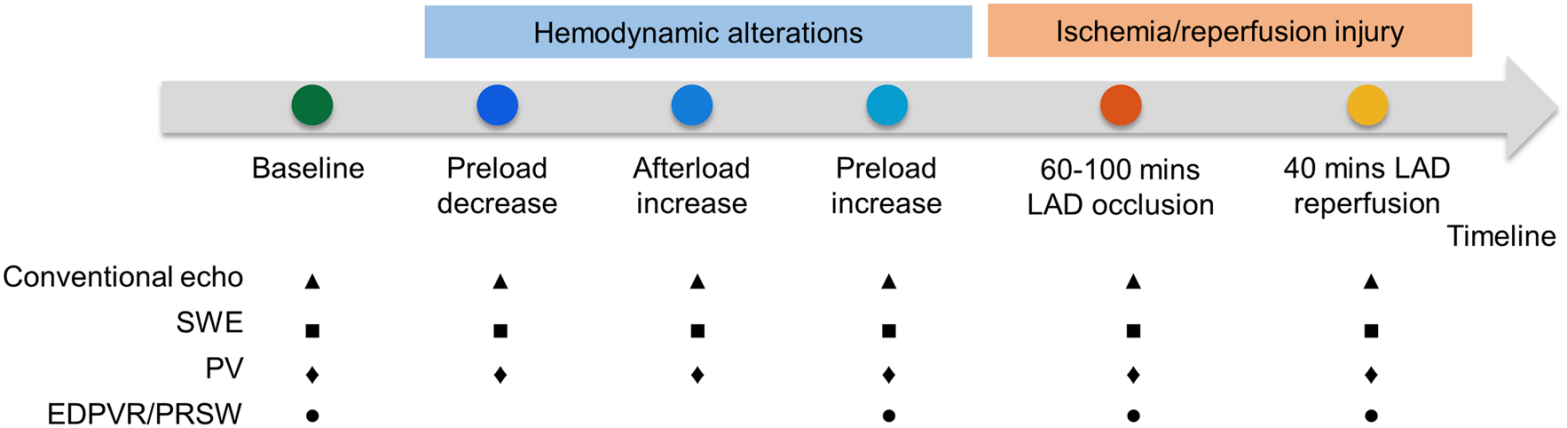
Timeline schematic for the pig experiments (SWE: shear wave elastography; PV: pressure–volume; EDPVR: end-diastolic pressure–volume relation; PRSW: preload-recruitable stroke work; LAD: left anterior descending artery).

The effects of hemodynamic alterations and an I/R injury on SWE and PV measurements in the pig model were investigated. Hemodynamic alterations entailed: (i) a decrease of preload by inflating the 8-F balloon catheter (PTS25, Braun Interventional system) by 2–5 ml in the vena cava inferior, (ii) an increase of afterload by inflating another 8-F balloon catheter by 2–5 ml in the descending aorta, and (iii) an increase of preload by rapidly administering 500 ml of saline intravenously. Measurements were taken when the pressure–volume loops stabilized. After the hemodynamic interventions, an I/R injury was induced by inflating a coronary angioplasty balloon (2.50 mm; Cordis) positioned in the proximal left anterior descending (LAD) coronary artery for 60–100 min. The coronary angioplasty balloon was then deflated and removed, and the tissue re-perfused for 40 min before the last measurement was performed. The timeline of these interventions is schematically shown in Fig. 1. During these interventions, multiple measurements were taken: conventional echocardiography (see Sect. 2.3), shear wave elastography (see Sect. 2.4), and pressure–volume measurements (see Sect. 2.5). Measurement of the end-diastolic pressure–volume relationship (EDPRV) and preload-recruitable stroke work (PRSW) were taken during inferior vena cava occlusion at an intervention stage (see Fig. 1).

### Conventional echocardiography

Left ventricular (LV) anatomy was quantified on B-mode images in the parasternal long-axis view using a GE Vivid E95 ultrasound scanner (GE Vingmed Ultrasound, Horten, Norway). More specifically, LV end-diastolic volume (EDV) and end-systolic volume (ESV) were derived from the measured LV diameter in the corresponding phase in the cardiac cycle using the Teichholz formula. These volumes are plugged into the volume calibration procedure of the pressure–volume analysis described in Sect. 2.5. Ejection fraction (EF) was subsequently derived from EDV and ESV. Septal wall thickness (IVSd) was calculated as the average of the measured wall thickness at basal and mid-ventricular level, corresponding to the region in which SWE measurements were performed. All measurements were performed using EchoPac software v202 (GE Healthcare, Horten, Norway).

### Shear wave elastography

#### Data acquisition

**Figure 2:**
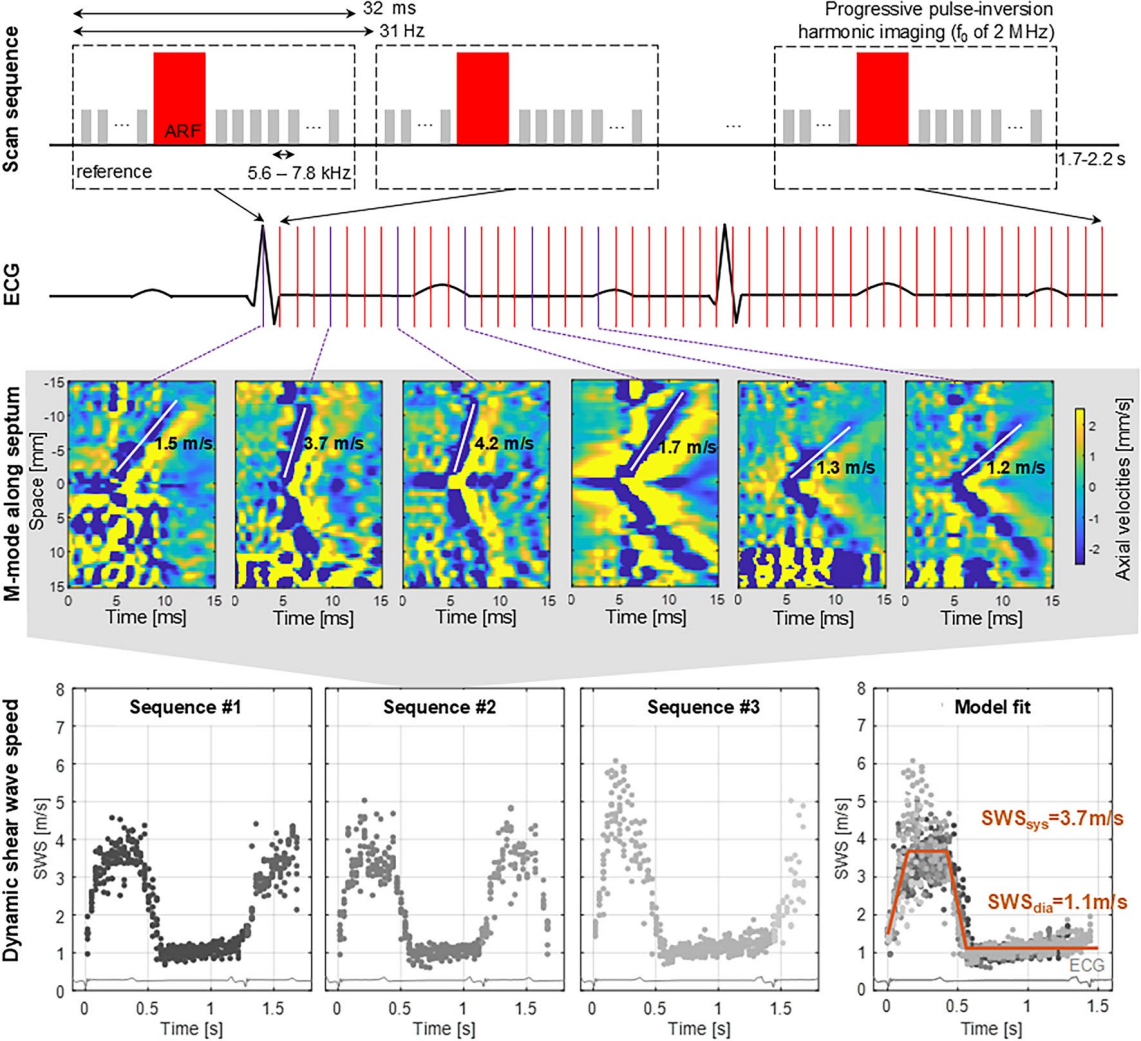
Shear wave elastography (SWE) sequence and postprocessing workflow. First row: schematic of a SWE imaging sequence, consisting of SWE acquisitions that were taken at 31 Hz during 1.7–2.2 s. Second row: schematic of an ECG signal. Third row: tissue velocity panels along the septum for different acquisitions at different time points, together with shear wave speed estimation. Fourth row: Shear wave speed data for three different SWE sequences at one intervention stage. The last panel demonstrates the procedure of obtaining diastolic and systolic shear wave propagation speed (SWS_dia_ and SWS_sys_) via piecewise linear model fitting. Different colors of grey represent different heartbeats. Spread of estimated wave speed at one time point represents variability across 10 anatomical M-lines drawn by the 2 observers.

Transthoracic SWE measurements were performed in a parasternal long-axis view with a Verasonics Vantage 256 ultrasound research system (Verasonics, Kirkland, United States). The first five pigs were scanned with a P4-2 phased array probe (ATL, Bothell, Washington, United States), whereas the other measurements were performed using a P4-2V phased array probe (Verasonics, Kirkland, United States). One SWE sequence consisted of 1.7–2.2 s recording time in which multiple individual SWE acquisitions were performed at intervals of 32 ms (31 SWE acquisitions per second), as illustrated in the first row of Fig. 2. The start of this sequence was triggered by the R-peak of the electrocardiogram (ECG; GWE 3-leads Cardiotachometer CT-1000, Ardmore, United States). The ECG signal was recorded with an external digital oscilloscope (TiePie Handyscope HS3 with multi-channel software, TiePie engineering, Sneek, the Netherlands), which also registered the timing of the SWE measurements based on trigger outputs of the Verasonics-scanner.

One SWE acquisition consisted of first acquiring 20 reference frames using ultrafast ultrasound, i.e. diverging wave imaging^11^. Second, a high-intensity focused ultrasound beam (center frequency of 2 MHz) was applied for 800 µs with a focal point depth set to the mid-septal wall. The resulting shear wave propagation was consecutively recorded at a minimal frame rate of 5.6 kHz using diverging wave imaging. Reference and tracking frames were acquired using a pulse-inversion harmonic transmission scheme (center frequency of 2 MHz) with a sliding window to maintain the frame rate. The radio frequency (RF) data together with the ECG signal were saved for offline processing.

#### Data analysis

Analytic in-phase and quadrature (IQ) image data were then obtained by offline beamforming the RF data using the Verasonics software in Matlab R2019a (Mathworks, Natick, MA, USA). A one-lag autocorrelator^12^ was subsequently used to compute the axial particle velocities in the IQ frames. To reduce the effect of noise, a Gaussian spatial smoothing filter of 1.9° by 2.0 mm was applied to the autocorrelation frames before taking the complex angle. Gross motion and high frequency noise were subsequently reduced by applying a 6th-order 75–750 Hz bi-directional Butterworth bandpass filter.

All SWE acquisitions were analyzed by 2 observers, who each manually drew 5 anatomical M-mode lines along the interventricular septum. The tissue velocity along that M-mode line was then plotted as a function of time, showing the wave propagation as a tilted pattern in this tissue velocity panel. The tilt angle represents the wave propagation speed, as shown in the third row of panels in Fig. 2. The wave propagation speed was determined manually by each observer, by indicating a start point and end point of a line following the wave front represented by the blue-colored band originating from the push location (as demonstrated by the white lines in Fig. 2), without any knowledge on the performed intervention. Automated analysis algorithms such as the Radon transform^13^ failed due to the limited signal-to-noise ratio. Resulting shear wave speed dynamics for three SWE sequences at one intervention stage is demonstrated in the last row of panels in Fig. 2. To determine systolic and diastolic wave propagation speed in a robust manner, a piecewise linear model consisting of four parts^14^—one positively inclined line, one horizontal line representing the systolic speed, one negatively inclined line and another horizontal line representing the diastolic speed (see Fig. 2)—was considered. This model was fitted in a non-linear least squares manner to the shear wave speed data measured between the two R-peaks of the ECG-signal. This procedure is demonstrated in Fig. 2: shear wave speed data of multiple sequences—representing different acquisitions, heartbeats and M-mode lines—were considered simultaneously to obtain the diastolic and systolic wave speed of an intervention. Next to diastolic and systolic wave speed, we also calculated the wave speed ratio^14^. To obtain a representative estimate of diastolic wave speed, systolic wave speed and speed ratio, a SWE sequence was only considered in the fitting procedure if shear waves were visible in > 20% of the SWE acquisitions.

### Pressure–volume recordingss

#### Data acquisition

For pressure and volume (PV) measurements, a 5-F pressure–volume Millar catheter (model MPR-500, Millar Instruments Inc., Houston, Texas) was placed in the LV under fluoroscopic guidance. Pressure, volume and ECG were registered at a sample rate of 1000 Hz using the MPVS Ultra Pressure–Volume loop acquisition system (Millar Instruments Inc.) and Labchart 7 Pro analysis software (AD instruments, Dunedin, New Zealand). For pressure calibration, the catheter was calibrated in a 37° water tank at the start of each experiment and its calibration was re-evaluated at the end of the experiment. For the total volume calculation, only the catheter-segments inside the left ventricle were considered, i.e. showing a counter-clockwise pressure–volume loop progression. Subsequently, a linear regression was performed between the voltages measured with the Millar catheter and the volumes measured with echocardiography in Sect. 2.3 considering end-diastole and end-systole of all interventions in one animal. This regression equation allowed to convert voltage data into volume data in a robust manner.

#### Data analysis

**Figure 3:**
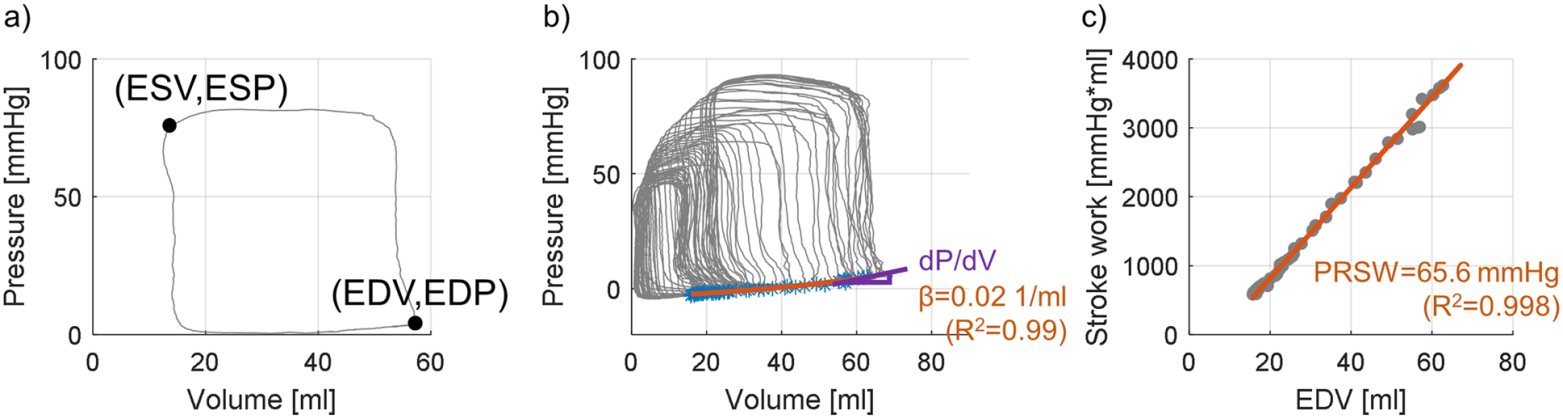
Quantities derived from the pressure–volume analysis. (**a**) End-diastolic and end-systolic pressure and volume. (**b**) End-diastolic pressure–volume relationship (EDPVR) fitting to obtain two measures of chamber stiffness: exponential coefficient β and local slope of the EDPVR (dP/dV). (**c**) Preload-recruitable stroke work (PRSW), i.e. the slope of a linear regression between EDV and stroke work, as measure for contractility.

The relevant cardiac cycles of the PV-data to analyze are selected based on the maximum of the cross-correlation function between the ECG of the PV analysis and the ECG of the SWE measurements. From the PV-data during these selected cardiac cycles, the following relevant parameters are extracted using Matlab R2021b (see also Fig. 3):

- *End-diastolic pressure (EDP):* pressure at onset of positive deflection of dP/dt (foot point of positive pulse with dP/dt_max_ in dP/dt-signal);
- *End-systolic pressure (ESP):* pressure for which the ratio of pressure and volume is maximal;
- *End-diastolic pressure–volume relationship (EDPVR):* an exponential relationship was assumed with the following equation^15^
  $$EDP = C \cdot \exp \left( {\beta \cdot \left( {EDV - D} \right)} \right) + E$$
  With end-diastolic pressure EDP, end-diastolic volume EDV and fitting constants C, β, D and E. The fitting constants are obtained by performing a nonlinear least squares fitting procedure considering the end-diastolic PV-data during preload reduction. We considered two measures of chamber stiffness: exponential coefficient β—often used to represent diastolic chamber properties^15^—and operating chamber stiffness dP/dV, i.e. the slope of the tangent to the EDPVR at (EDV, EDP).
- *Preload recruitable stroke work (PRSW):* the slope of a robust linear regression between stroke work (SW) and EDV during preload reduction gives information about contractility. PRSW provides a load-independent measure of the contractility of the heart, and has shown to be a more robust and clinically useful index than end-systolic elastance^16, 17^.

### Statistical analysis

Three SWE sequences were captured for one specific intervention, and an average value together with its standard deviation is reported. Hemodynamic, echocardiographic, PV and SWE data between different interventions were compared using a paired Student’s t-test with Bonferroni correction for repeated measures. Linear regression and Pearson’s correlation coefficient R were calculated to assess the correlation between two variables. A p-value less than 0.05 was considered statistically significant. All statistical analyses were performed in Matlab R2021b.

## Results

### Clinical parameters

One pig died during LAD occlusion due to fatal arrhythmia. The effect of hemodynamic interventions and myocardial I/R injury on clinical, hemodynamic and pressure–volume data is summarized in Table 1. From all variables, only heart rate did not significantly change across these interventions.

**Table 1:** Clinical, hemodynamic and pressure–volume data (HR: heart rate, IVSd: septal wall thickness, LV EDV: left ventricular end-diastolic volume, LV ESV: left ventricular end-systolic volume, EF: ejection fraction, EDP: end-diastolic pressure, ESP: end-systolic pressure, PRSW: preload-recruitable stroke work, β: exponential coefficient of the end-diastolic pressure–volume relationship, dP/dV: operational stiffness). Statistical significant changes with respect to baseline are typeset in bold and indicated with an asterisk.

	Baseline	Preload decrease	Afterload increase	Preload increase	Ischemia	Reperfusion
HR (bpm)	70 ± 24	66 ± 27	55 ± 13	64 ± 12	70 ± 13	98 ± 27
IVSd (mm)	9 ± 1	10 ± 1*	9 ± 1	9 ± 1	9 ± 1	13 ± 2*
LV EDV (ml)	73 ± 11	44 ± 16*	82 ± 8*	85 ± 11*	76 ± 7	57 ± 13
LV ESV (ml)	29 ± 5	16 ± 6	38 ± 6*	31 ± 6	36 ± 8	26 ± 9
EF (%)	60 ± 3	60 ± 6	56 ± 3*	64 ± 3	55 ± 10	58 ± 7
EDP (mmHg)	5 ± 3	− 4 ± 2*	11 ± 5	9 ± 4	16 ± 3*	11 ± 10
ESP (mmHg)	98 ± 17	48 ± 9*	122 ± 17*	103 ± 16	85 ± 10	70 ± 7
PRSW (mmHg)	53 ± 12	–	–	54 ± 9	36 ± 13	24 ± 7*
β (1/ml)	0.045 ± 0.013	–	–	0.060 ± 0.025	0.073 ± 0.020	0.087 ± 0.030*
dP/dV (mmHg/ml)	0.49 ± 0.18	0.07 ± 0.04*	0.69 ± 0.13*	0.87 ± 0.34*	1.41 ± 0.25*	1.32 ± 0.70*

The hemodynamic interventions significantly altered end-diastolic pressure (EDP of 5 mmHg at baseline) with − 172% during preload decrease, resulting in negative pressures. This can be expected from a severe preload reduction in healthy hearts, where the blood is sucked into the ventricle. The end-diastolic volume (EDV of 73 ml at baseline) significantly changed during preload decrease (− 40%), afterload increase (+ 12%) and preload increase (+ 16%). Loading changes also significantly affected end-systolic pressure (ESP of 98 mmHg at baseline) during preload decrease (− 51%) and afterload increase (+ 25%); and end-systolic volume (ESV of 29 ml at baseline) during afterload increase (+ 31%). All changes in operational chamber stiffness dP/dV during loading interventions were significant: − 86%, + 41% and + 78% during preload decrease, afterload increase and preload increase, respectively.

The I/R injury increased myocardial stiffness: the exponential coefficient of the EDPVR (β) increased by 62% after the ischemia period and further increased by 93% (*p* < 0.05) after the reperfusion period. This corresponded with a significant operational chamber stiffness dP/dV change of + 188% and + 169% after the infarction and reperfusion phase, respectively. The I/R injury also resulted in a significantly thicker septal wall (+ 44% in IVSd), and a significant lower contractility (− 54% in PRSW).

### Feasibility and variability of SWE measurements

**Figure 4:**
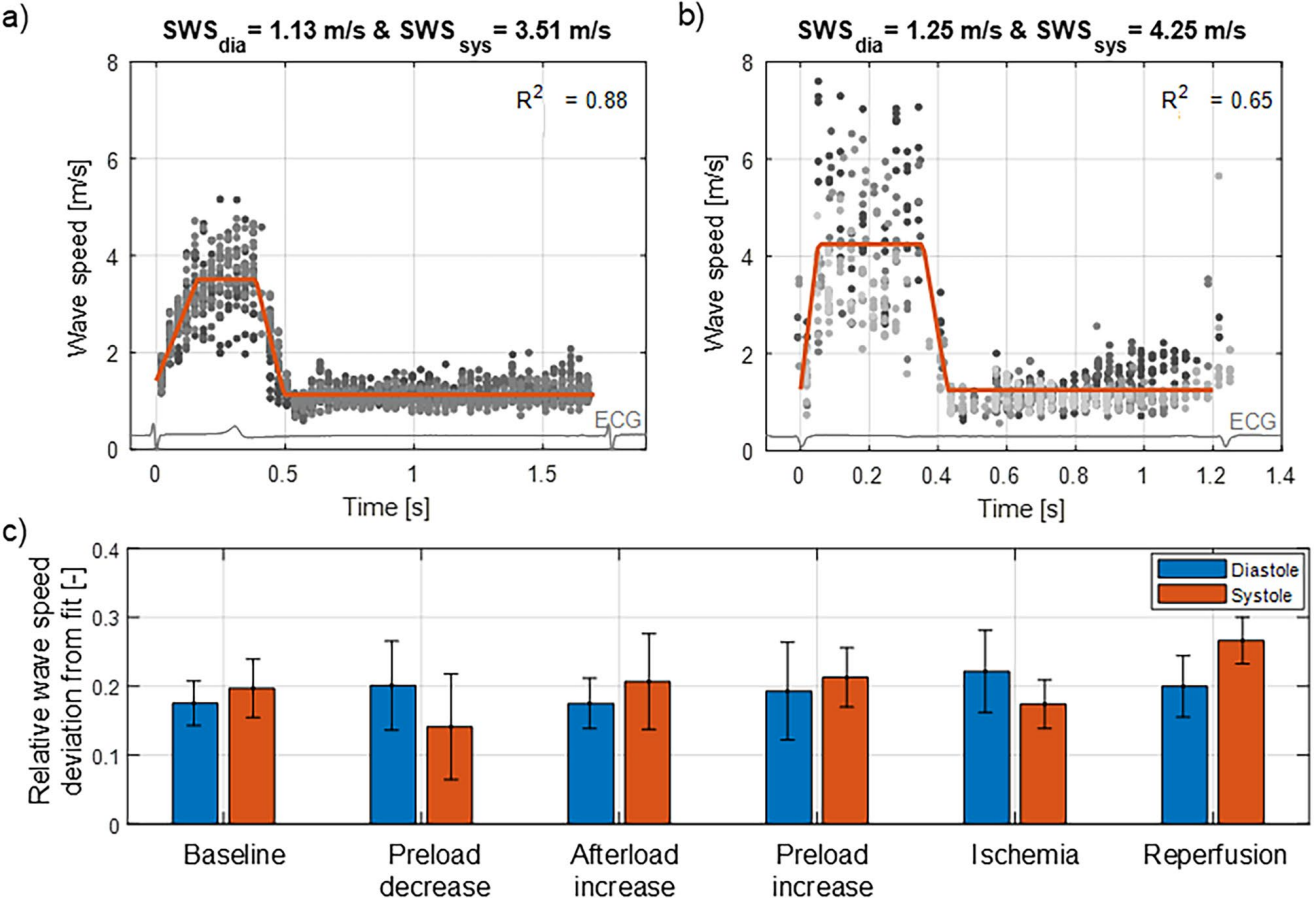
Variability of shear wave speed (SWS) estimation. (**a**) Example of low variability and good fit. (**b**) Example of high variability and moderate fit. (**c**) Averaged relative wave speed deviation from fit for all pigs is depicted for each condition and diastole/systole.

SWE measurements were successful in 7 out of the 13 pigs (success rate of 54%). In these 7 pigs, 93 of the 126 SWE sequences (74%) were considered for further analysis. Representative examples of shear wave speed variability in an SWE sequence due to the observer, heartbeat and anatomic M-mode line placement are shown in Fig. 4a, b, where each dot represents one manual wave propagation speed estimate. The average relative wave speed deviation with respect to the fitted diastolic and systolic wave speed (SWS_dia_ and SWS_sys_) from the piecewise linear model fit is depicted in Fig. 4c for each intervention. Wave speed variability in diastole and systole are similar (19.4% vs. 20.0% on average for the different interventions).

### Diastolic wave speed

**Figure 5:**
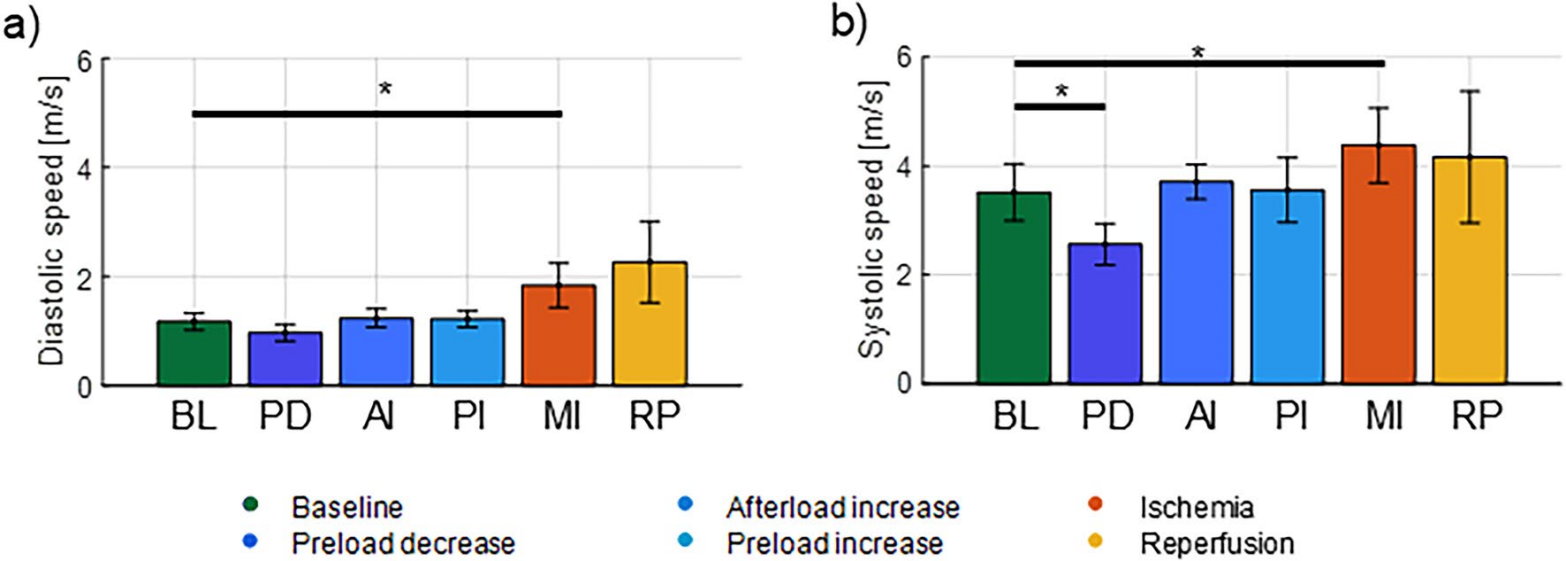
Diastolic and systolic wave speeds for the different interventions: baseline (BL), preload decrease (PD), afterload increase (AI), preload increase (PI), myocardial ischemia (MI) and reperfusion (RP). *p < 0.05 for t-test with Bonferroni correction.

The diastolic wave speed for the different interventions is summarized in Fig. 5a, with a wave speed of 1.2 m/s in baseline. Significant changes in wave speed were only observed after ischemia injury (+ 57), whereas other interventions did not significantly alter the wave speed (− 17% in preload decrease, + 5% in afterload increase, + 4% in preload increase and + 94% after reperfusion).

**Figure 6:**
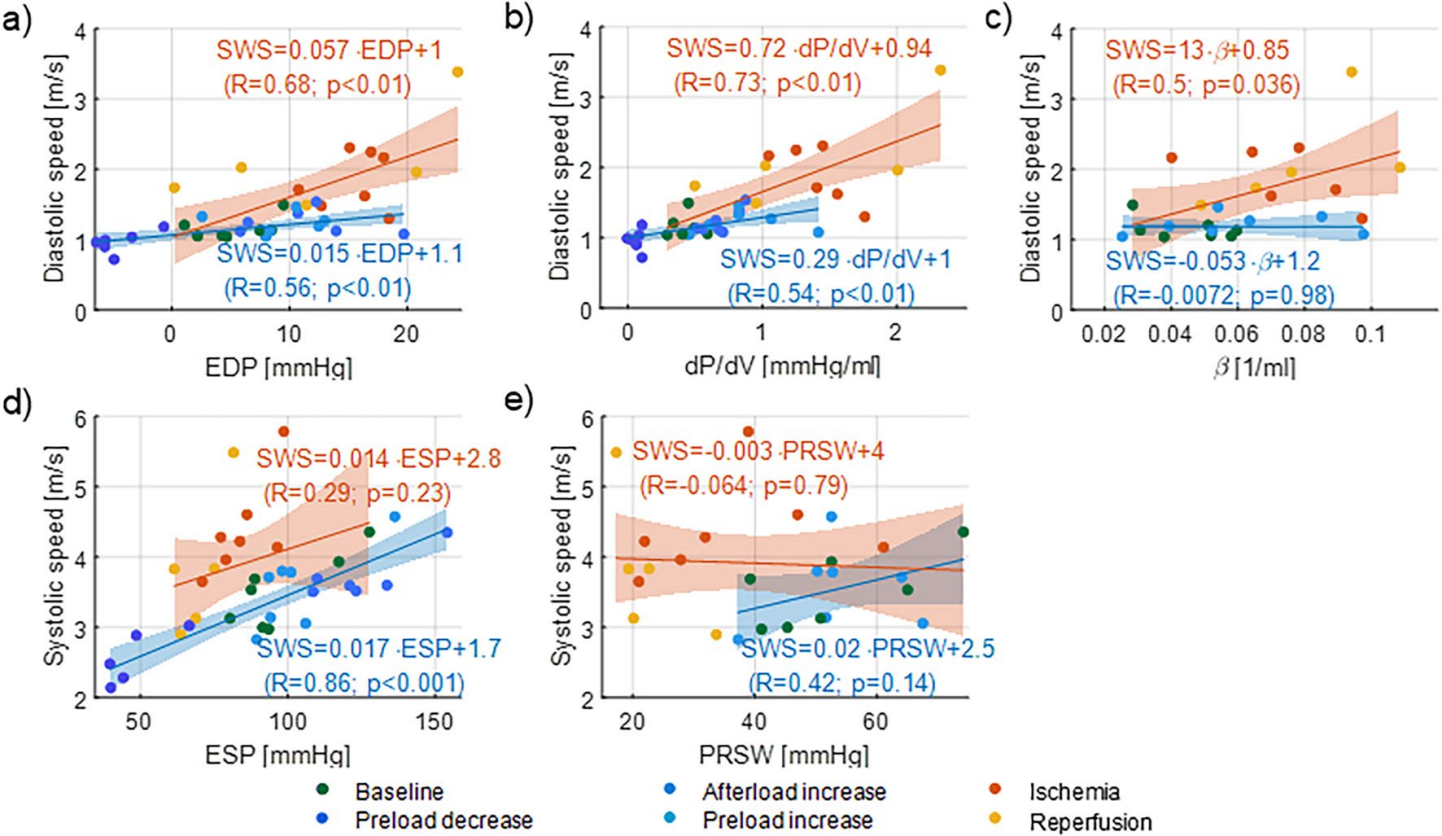
Correlations of diastolic and systolic wave speed, with (**a**) end-diastolic pressure (EDP), (**b**) operational stiffness (dP/dV), (**c**) stiffness constant β, (**d**) end-systolic pressure (ESP) and (**e**) preload-recruitable stroke work (PRSW) during loading (blue) and stiffness interventions (orange).

Diastolic wave speed is significantly correlated to EDP when altering the loading (blue in Fig. 6a: R = 0.56; *p* < 0.01). The linear regressions during the loading interventions are tabulated in Table 2 for each pig, showing a good reproducibility between the animals. A similar correlation is found between SWS and operational chamber stiffness dP/dV (R = 0.54; *p* < 0.01 in Fig. 6b). SWE measurements during and after I/R injury—as depicted in orange in Fig. 6—showed a strong significant correlation to EDP (R = 0.68; *p* < 0.01), operational chamber stiffness dP/dV (R = 0.73; *p* < 0.01) and stiffness constant β R = 0.50; *p* = 0.03). Diastolic wave speed is more sensitive to changes in intrinsic stiffness than in loading, as reflected by the larger slope of the regression line (0.73 vs. 0.29 in Fig. 6b).

**Table 2:** Linear regression results between diastolic wave speed (SWS_dia_) and end-diastolic pressure (EDP) on one hand and systolic wave speed (SWS_sys_) and end-systolic pressure (ESP) on the other hand during loading interventions, with goodness of fit R^2^.

Pig #	SWS_dia_ versus EDP			SWS_sys_ versus ESP		
	Slope (m/s/mmHg)	Intercept (m/s)	R^2^	Slope (m/s/mmHg)	Intercept (m/s)	R^2^
1	0.010	1.0	0.92	0.017	1.4	0.93
2	0.024	1.2	0.92	0.016	1.5	0.99
3	0.015	1.0	0.93	0.021	1.2	0.95
4	0.019	1.0	0.53	0.023	1.6	0.997
5	0.017	1.1	0.60	0.011	2.5	0.85
6	0.012	1.0	0.81	0.021	1.5	0.91
7	0.013	1.1	0.70	0.017	2.0	0.94
Mean	0.016 ± 0.005	1.1 ± 0.1	0.77 ± 0.17	0.018 ± 0.004	1.7 ± 0.4	0.94 ± 0.05

### Systolic wave speed

Figure 5b shows the resulting systolic wave speed for all interventions, with a wave speed of 3.5 m/s at baseline. The wave speed altered significantly during preload decrease (− 27%) and after ischemia injury (+ 25%).

Systolic wave speed significantly correlated with only one measure of contractility during loading interventions: ESP in Fig. 6d (R = 0.86; *p* < 0.001) and not PRSW in Fig. 6e. The individual correlations with ESP are given in Table 2, and show in general a slightly higher goodness-of-fit (R^2^) than for the diastolic measurements, probably due to the limited sensitivity of the manual wave speed estimator to detect differences in speed (ΔSWS_dia_ = 0.33 m/s vs. ΔSWS_sys_ = 1.14 m/s). No significant correlation was found between systolic wave speed and measures of contractility during I/R injury.

### Wave speed ratio

**Figure 7:**
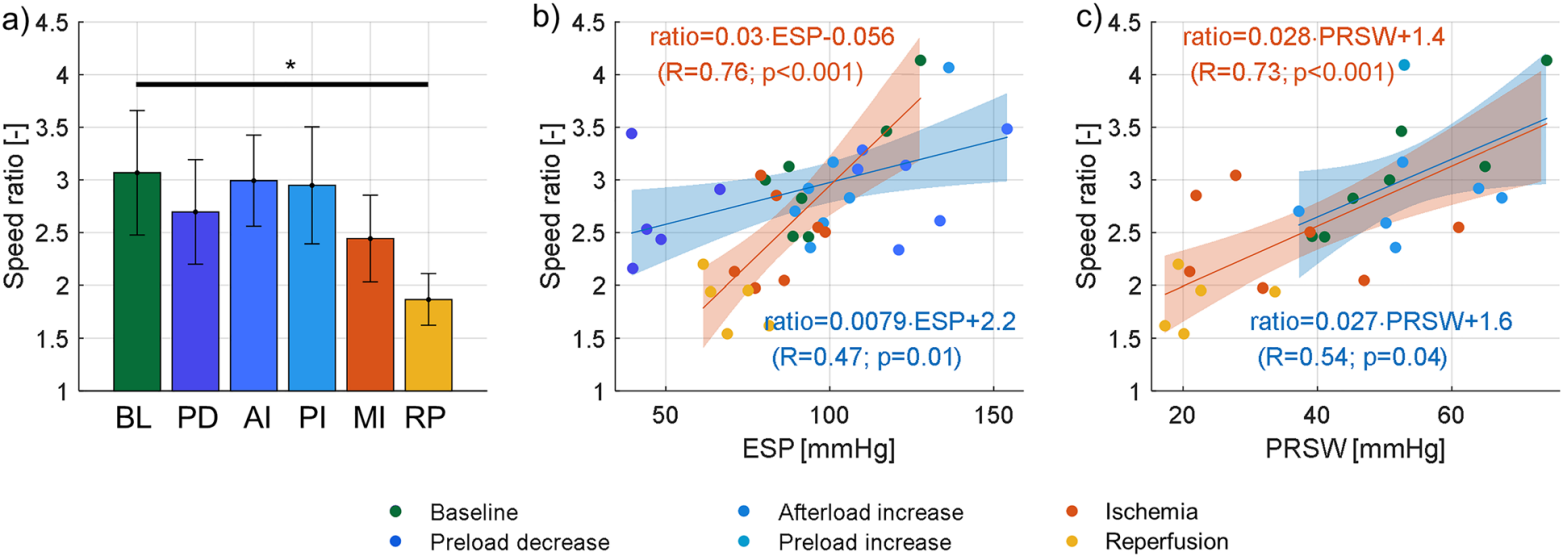
Wave speed ratio results (ratio of systolic and diastolic wave speed). (**a**) Wave speed ratio for the different interventions: baseline (BL), preload decrease (PD), afterload increase (AI), preload increase (PI), myocardial infarct (MI) and reperfusion (RP). *p < 0.05 for t-test with Bonferroni correction. (**b**) Correlation between wave speed ratio and end-systolic pressure (ESP). (**c**) Correlation between wave speed ratio and preload-recruitable stroke work (PRSW).

The ratio of the systolic and diastolic wave speed for the different interventions is depicted in Fig. 7, together with its correlation to measures of contractility. The speed ratio was 3.07 at baseline and did not significantly alter during loading interventions (− 11% in preload decrease, − 1% in afterload increase and − 2% in preload increase). The I/R injury caused a significant decrease of 38%.

During loading interventions, a moderate statistically significant correlation was found between the speed ratio and ESP (R = 0.47; *p* = 0.017 in Fig. 7b), and the speed ratio and PRSW (R = 0.54; *p* = 0.044 in Fig. 7c). The correlation between wave speed ratio and measures of contractility was stronger for the I/R injury: R = 0.76 with ESP in Fig. 7b and R = 0.73 for PRSW in Fig. 7c.

## Discussion

To the best of the authors’ knowledge, this study is the first to compare transthoracic shear wave elastography (SWE) recordings throughout the entire cardiac cycle to invasive pressure–volume (PV) measurements for evaluating the myocardial stiffness dynamics in vivo. A moderate feasibility of the SWE-method was shown (success rate of 54%) and wave speed estimations varied 19.4% in diastole and 20.0% in systole on average. By performing a robust estimate of wave speed, this study linked diastolic wave speed to a pressure–volume loop-derived index of operational myocardial stiffness, i.e. dP/dV. Operational myocardial stiffness takes into account the stiffening effect due to increased ventricular pressure, stressing the importance of hemodynamic loading for diastolic wave speed. The ratio of systolic and diastolic wave speed correlated to pressure–volume loop-derived indices of contractility, i.e. end-systolic pressure (ESP) and preload-recruitable stroke work (PRSW). Measuring this ratio might thus provide a non-invasive index of contractility during I/R injury.

### Feasibility of transthoracic SWE

The feasibility of SWE depends on three important factors: the amplitude of the acoustically induced vibrations, the quality of the images in which the vibrations are tracked, and the quality of the tracking algorithm itself to cope with underlying tissue motion. Transthoracic application of SWE to the heart poses extra difficulties as (i) cardiac phased array probes have a relatively small footprint and thus limited excitation strength and image quality, and (ii) cardiac tissue is moving relatively fast, challenging tracking algorithms but also push beam settings for powerful shear wave excitation throughout the cardiac cycle. This explains in part our moderate success rate of 54% (7/13 pigs). Some of the SWE acquisitions in the other 6 ‘unsuccessful’ pigs did reveal shear wave propagation but were not included in our analysis as there were only consistent SWE results for maximal 2 out of the 6 interventions. Especially at the start of our pig experiment, there were more ‘unsuccessful’ pigs as the experimental set-up gradually improved (use of a new generation probe and repositioning of the pigs at the beginning to obtain the best possible B-mode image quality in terms of contrast and SNR). Future work should investigate the feasibility of SWE in a systematic manner while varying the set-up and sequence settings, but this was outside the scope of this work. For example, the effect of using a more accurate but computationally more expensive tissue motion estimator than Kasai’s algorithm (e.g. Loupas’algorithm^18^) needs to be studied in the future.

Our reported feasibility is higher than the success rate of our dynamic SWE experiments in open-chest pigs^19^, where a success rate of 32% was reported. It is also slightly higher than the success rate of 41% reported by Kakkad et al.^20^, who used another technology (acoustic radiation force based imaging) to investigate qualitatively the dynamic stiffness variations across the cardiac cycle. The success rate increases tremendously when applying SWE at one time point during the cardiac cycle (end-diastole) according to previous clinical studies: 77.5% in healthy children^21^ and 91.3% in healthy adults^22^. Recently, some preliminary results have been reported on the dynamic stiffness variations in healthy children, but feasibility rate of these measurements is currently unknown^23^. Technical improvements are currently ongoing to increase the feasibility of SWE: optimization of filters to suppress background motion^20^, improvements in the imaging sequence, such as ultrafast harmonic coherent compound imaging^24^, or development of a new ultrasound transducer with a better mechanical focus for shear wave excitation^25^.

Next to the challenges to induce and observe the shear wave in the myocardium, there are also challenges to provide a robust estimate of the wave speed due to its variability (averaged variability of ± 20% in systole and diastole in this study, whereas the limit of agreement in ideal conditions is 0.1 m/s^26^). The variability in wave speed estimation might arise from the dependence of the wave propagation on the ventricular morphology, the stress state (ventricular pressures) and the myocardial fiber orientation^27^. Furthermore, different acquisitions or heartbeats contribute to the natural physiological variability of the wave speed. Also, experimental settings such as the selected protocol, the number of observers, and the selected wave speed estimator will affect wave speed estimation. We accounted for part of this variability by performing wave speed estimation for the total 10 M-mode lines across the septal wall drawn by 2 observers and subsequently fitting a piecewise linear model to the speed data of multiple heartbeats and acquisitions (see Fig. 2). Wave speed estimation was performed manually, which was time-consuming and subjective (despite our blinded analysis), and should be automated in future work by using for example an approach with masking functionality to remove noisy regions as recently proposed by Jin et al.^28^ Furthermore, we believe that combining the linear regression fit with automation of wave speed estimation and M-mode line positioning are essential for an accurate and robust wave speed estimation, but also for clinical implementation.

### Myocardial operational stiffness

Diastolic wave speed has been suggested as measure for myocardial stiffness by several studies through estimating the significance level of an infarcted vs. non-infarcted group in comparison to the significance of changes in other stiffness parameters: the stiffness constant of the end-diastolic stress–strain relation^6, 7^ and the end-diastolic pressure–volume relation (EDPVR)^5^. Our study confirms this earlier research: the change in the stiffness constant β of the EDPVR increased after the ischemia period (0.073 vs. 0.045 1/ml; *p* = 0.06) and increased even further after the reperfusion period (0.087 vs. 0.045 1/ml; *p* < 0.05), which was reflected in the change of diastolic wave speed after ischemia injury (1.8 vs. 1.2 m/s; *p* < 0.05) and reperfusion injury (2.3 m/s). Furthermore, our work showed a significant correlation between diastolic wave speed and stiffness constant β in Fig. 6c (R = 0.50; *p* = 0.04). Literature does not report any correlations between diastolic wave speed and stiffness parameters, however a clinical study in hypertrophic cardiomyopathy patients^22^ showed a significant correlation between diastolic wave speed and fibrosis markers in cardiac magnetic resonance: T1 post-contrast (R = 0.595; *p* = 0.01) and late gadolinium enhancement (R = 0.80; *p* < 0.01).

**Figure 8:**
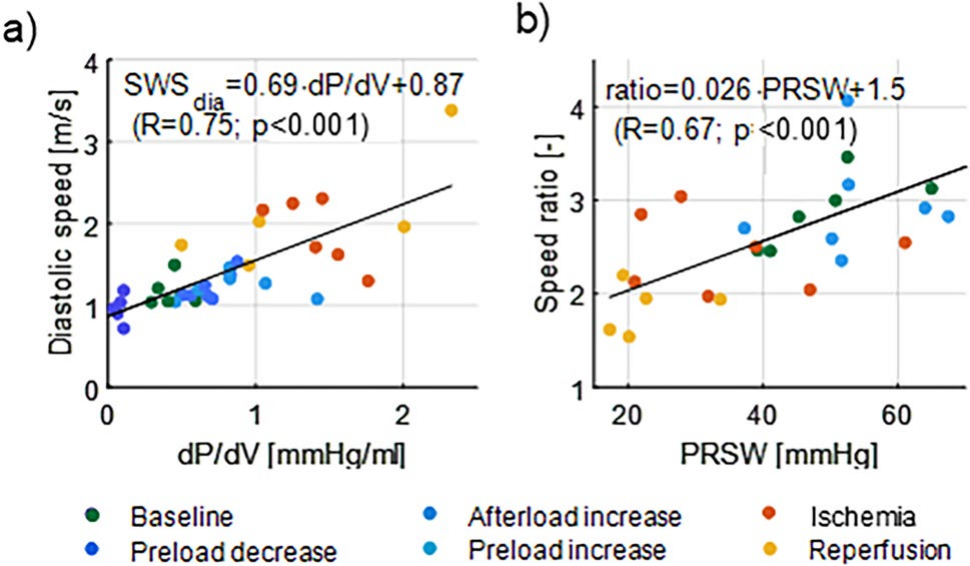
Correlation between diastolic wave speed and operational chamber stiffness (dP/dV) and between wave speed ratio and preload-recruitable stroke work (PRSW) for all interventions.

The exponential coefficient β of the EDPVR is typically considered an index for diastolic chamber stiffness^15^, however it does not provide insights into the stiffness dynamics. Operational chamber stiffness, where a change in pressure is considered relative to a change in volume (or dP/dV), allows to assess the local slope of the EDPVR at a specific filling pressure and thus increases when pressure increases due to the non-linear ESPVR. Therefore, we considered dP/dV next to stiffness constant β in this study in order to describe any stiffness changes due to loading and/or intrinsic stiffness alterations. As tabulated in Table 1, all interventions—hemodynamic or I/R injury—significantly altered the operational stiffness dP/dV. Diastolic wave speed significantly altered only after ischemia injury (see Fig. 5). It did show a significant positive correlation with dP/dV for both types of interventions (R = 0.57; *p* < 0.01 for loading and R = 0.68; *p* < 0.01 for stiffness interventions). Furthermore, the correlation was even stronger when considering all interventions (R = 0.75; *p* < 0.001 in Fig. 8a). This highlights that diastolic wave speed depends on ventricular loading and intrinsic stiffness and thus rather measures *operational* myocardial stiffness. The difference in the slopes of the fitted linear regression curves in Fig. 6b (0.73 vs. 0.29) suggests that diastolic speed is more sensitive to changes in intrinsic characteristics than changes in loading. This would be beneficial for clinical evaluation of intrinsic myocardial properties, or evaluation of loading if changes in stiffness can be ruled out based on the patient profile.

The results of our statistical analysis on the group level suggests a preload independence of diastolic wave speed, which is in correspondence with other studies^2, 7^, but our linear regression results demonstrate that loading does have an effect on diastolic wave speed, but to a lesser extent than the intrinsic properties. These observations can be partly explained by differences in the considered pressure change amongst studies (largest pressure drop in this study, i.e. 26.1 mmHg, but in the same range as observed in diseased human hearts^29^), and the low sample size of our study. Furthermore, the effect of material nonlinearity can also be observed from the changes of the wave speed as a function of time during diastole in Figs. 2 and 4a, b, showing an increasing trend towards end-diastole for some heartbeats. Further work in a larger population is however necessary to verify these findings. A true load-independent measure of myocardial stiffness might be obtained by looking at the slope of the linear fit between diastolic wave speed and EDP during loading interventions (see Table 2), demonstrating a good reproducible fit for each pig. It still needs to be investigated whether (i) this slope alters during I/R injury, and (ii) a non-invasive loading change such as leg lifting can induce a change in wave speed that can be picked up. As an elevated pressure typically coincides with an elevated myocardial stiffness in cardiac disease, distinguishing a wave speed increase due to elevated pressure or stiffness might offer additional insights into pathophysiology.

Next to loading, wall thickness may also affect the measured wave propagation speed in thin-walled media, as the wave might become guided due to the thin walls. When considering an analytical model describing wave propagation in a plate, i.e. a Lamb wave, an increased thickness can result in an increased wave speed for a specific frequency (main excited frequency of ± 150 Hz as discussed in the Supplementary information) and stiffness, which could provide an alternative explanation of the diastolic wave speed increase during MI. It is again difficult to attribute the increase in wave speed during MI rather to wall thickness (see IVSd in Table 1) than stiffness (see stiffness constant β of the EDPVR in Table 1) or vice versa, as both altered significantly. However, we believe the guiding effect in the current study is minimal as the wall thickness (9 mm in baseline, see Table 1) is larger than (i) the wavelength (± 6.5 mm on average in Fig. 2), which is the typical criterion for the existence of guided waves, and (ii) the recently presented cut-off of 4.4 mm (age > 1 month) by Malik et al.^30^ for which diastolic speed no longer depended on wall thickness in a multivariate linear analysis for their (unreported) shear wave frequencies.

### Contractility

Systolic wave speed has been put forward as a measure for contractility in literature, because of its excellent correlation with ESP during isoproterenol stimulation^2^ and with coronary perfusion pressure via the Gregg effect^31^. The current study characterized contractility using two parameters, i.e. ESP and loading-independent PRSW. The results showed that systolic wave speed reflected the changes in contractility during the loading interventions (due to the Frank-Starling principle), as can be seen from the strong correlation with ESP in c. However, systolic wave speed increased significantly after ischemia injury (4.4 vs. 3.5 m/s; *p* = 0.01 in Fig. 6c), which does not correspond with the observed decline in contractility in terms of pressure–volume measures after the I/R injury (see Table 1). Also, no correlation was found between systolic wave speed and any measure of contractility during I/R injury (see Fig. 6c, d). In literature, contradictory results are found concerning systolic wave speed changes after I/R injury: decrease after 20 min ligation of the LAD^4^ and (non-significant) increase after 1–2 h ligation of the LAD^7^.

In theory, an acute myocardial infarction is associated with an elevated myocardial stiffness and decreased contractility^32^. We hypothesized that both mechanisms affect systolic wave speed in an opposing manner, and it is therefore unsure what the net resulting effect is. This study therefore investigated the ratio of the systolic and diastolic wave speed as potential index of contractility: the wave speed ratio moderately correlated with measures of contractility for loading interventions (R = 0.47; *p* = 0.02 for ESP and R = 0.54; *p* = 0.04 for PRSW in Fig. 7b, c), whereas a strong correlation was found for the stiffness interventions (R = 0.76; *p* < 0.001 for ESP and R = 0.73; *p* < 0.001 for PRSW in Fig. 7b, c). Furthermore, the equation of the linear regression line between wave speed ratio and PRSW was almost identical for loading and stiffness interventions, and resulted in $\frac{{SWS_{sys} }}{{SWS_{dia} }} = 0.028 \cdot PRSW + 1.4$ (R = 0.6; *p* < 0.001) when considering both type of interventions simultaneously in Fig. 8b. This demonstrates the same sensitivity of wave speed ratio to both types of interventions.

The sensitivity of the use of wave speed ratio as non-invasive index of contractility needs to be further investigated in a larger clinical study as the current study only explored a limited number of animals (n = 7) and the induced changes in contractility are larger than what is reported in cardiac disease, e.g. PRSW altered + 17.5% in hypertension and − 6.9% in heart failure with preserved ejection fraction^33^. Furthermore, it should be noted that the same considerations as for diastolic wave speed measurements are valid concerning the effect of loading and wall thickness.

### Study limitations and future work

It should be noted that SWE assesses systolic and diastolic ventricular properties in a local region (~ 3 cm) of the septal wall, whereas pressure–volume loop analysis provides functional measures of the left ventricular chamber as a whole. In this study, interventions were chosen such that they resulted in global alterations of left ventricular function or in local (septal) changes via LAD occlusion. Therefore, the local SWE measurements in the septal wall strongly correlated with the global pressure–volume measures. SWE measurements in different regions and walls of the heart can offer us insights into the regional differences of systolic and diastolic ventricular properties.

PRSW and EDPVR were not measured during preload decrease and afterload increase as this required insertion of a second balloon in the vena cava inferior and execution of two interventions at the same time. Also, right ventricular pressure was not measured even though this pressure also affects the resulting stresses and strains in the septal wall, and consequently wave speed. It is however unsure how much right ventricular pressure alters in case of an acute septal infarct.

In this study, the wave propagation speed reported is rather a group speed, as it represents the speed of a pulse comprising multiple frequencies (50–500 Hz), but this does not strictly align with the textbook-definition of group speed^34^, for which an analysis in the Fourier domain is necessary. This type of analysis was not possible in current work due to limited SNR, but should ideally be considered in future work. We refrained from converting the wave propagation speed value into a stiffness value, because the myocardium is not an unbounded homogeneous isotropic linear elastic medium, which is required for the well-known conversion in SWE.

As current study presented animal experiments, SWE measurements did not fulfill the FDA guidelines concerning acoustic safety for human exams (MI = 2.2 and I_sppa,0.3_ = 403 W/cm^2^ for a push beam with a focal depth of 60 mm and push voltage of 60 V). Acoustic safety can however be guaranteed through ECG gating or programming scanner off time after every SWE acquisition to reduce the spatial peak temporal average acoustic intensity. Acoustic safety of dynamic shear wave measurements has recently been demonstrated by Malik et al.^23^ in healthy volunteers and hypertrophic cardiomyopathy patients. Concerning SWE data processing, diastolic and systolic wave speeds were now derived from a piecewise linear model, as suggested by Hollender et al.^14^, but this model might need to be tuned to account for higher end-diastolic wave speeds (see Fig. 4).

## Conclusions

This work related diastolic and systolic wave speed to pressure–volume loop derived indices of loading, myocardial stiffness and contractility in a closed-chest pig model. Diastolic wave speed was linked to operational myocardial stiffness, with a larger sensitivity to changes in intrinsic stiffness than in loading. The ratio of systolic and diastolic wave speed was related to measures of contractility. Our findings indicate that shear wave elastography might provide a non-invasive alternative to pressure–volume recordings in order to map the cardiac stiffness dynamics in vivo.

## Supplementary Information

Supplementary Information.

## Data Availability

The datasets for this study are available from the corresponding author on reasonable request.
