# Correction: Continuous shear wave measurements for dynamic cardiac stiffness evaluation in pigs

**DOI:** 10.1038/s41598-026-56687-z

**Published:** 2026-06-08

**Authors:** Annette Caenen, Lana Keijzer, Stéphanie Bézy, Jürgen Duchenne, Marta Orlowska, Antonius F. W. Van Der Steen, Nico De Jong, Johan G. Bosch, Jens‑Uwe Voigt, Jan D’hooge, Hendrik J. Vos

**Affiliations:** 1 Department of Cardiology, Erasmus MC University Medical Center, Rotterdam, The Netherlands; 2 Cardiovascular Imaging and Dynamics Lab, KU Leuven, Leuven, Belgium; 3 Institute for Biomedical Engineering and Technology, Ghent University, Ghent, Belgium; 4 Department of Imaging Physics, Delft University of Technology, Delft, The Netherlands; 5 Cardiology, KU Leuven, Leuven, Belgium

Correction to: *Scientific Reports* 10.1038/s41598-023-44588-4, published online 17 October 2023

The original version of this Article contained errors.

An error in the pulse repetition frequency (PRF) of the imaging sequence was discovered during subsequent analysis of the data. Acoustic verification of the original acquisition script confirmed the incorrect PRF. Although the relative comparisons in the study remain valid because the PRF error was the same across all conditions, the absolute wave speed values require correction with a factor 0.901 to ensure scientific accuracy. As the result, in Materials and methods section, under ‘Shear wave elastography’ subheading,

“One SWE sequence consisted of 1.5 - 2 s recording time in which multiple individual SWE acquisitions were performed at intervals of 28 ms (34 SWE acquisitions per second), as illustrated in the first row of Fig. 2.”

now reads:

“One SWE sequence consisted of 1.7 - 2.2 s recording time in which multiple individual SWE acquisitions were performed at intervals of 32 ms (31 SWE acquisitions per second), as illustrated in the first row of Fig. 2.”

“The resulting shear wave propagation was consecutively recorded at a minimal frame rate of 6.2 kHz using diverging wave imaging.”

now reads:

“The resulting shear wave propagation was consecutively recorded at a minimal frame rate of 5.6 kHz using diverging wave imaging.”

As the result, Figure 2 and its legend were incorrect. The original Figure 2 and accompanying legend appear below.

**Fig. 2:**
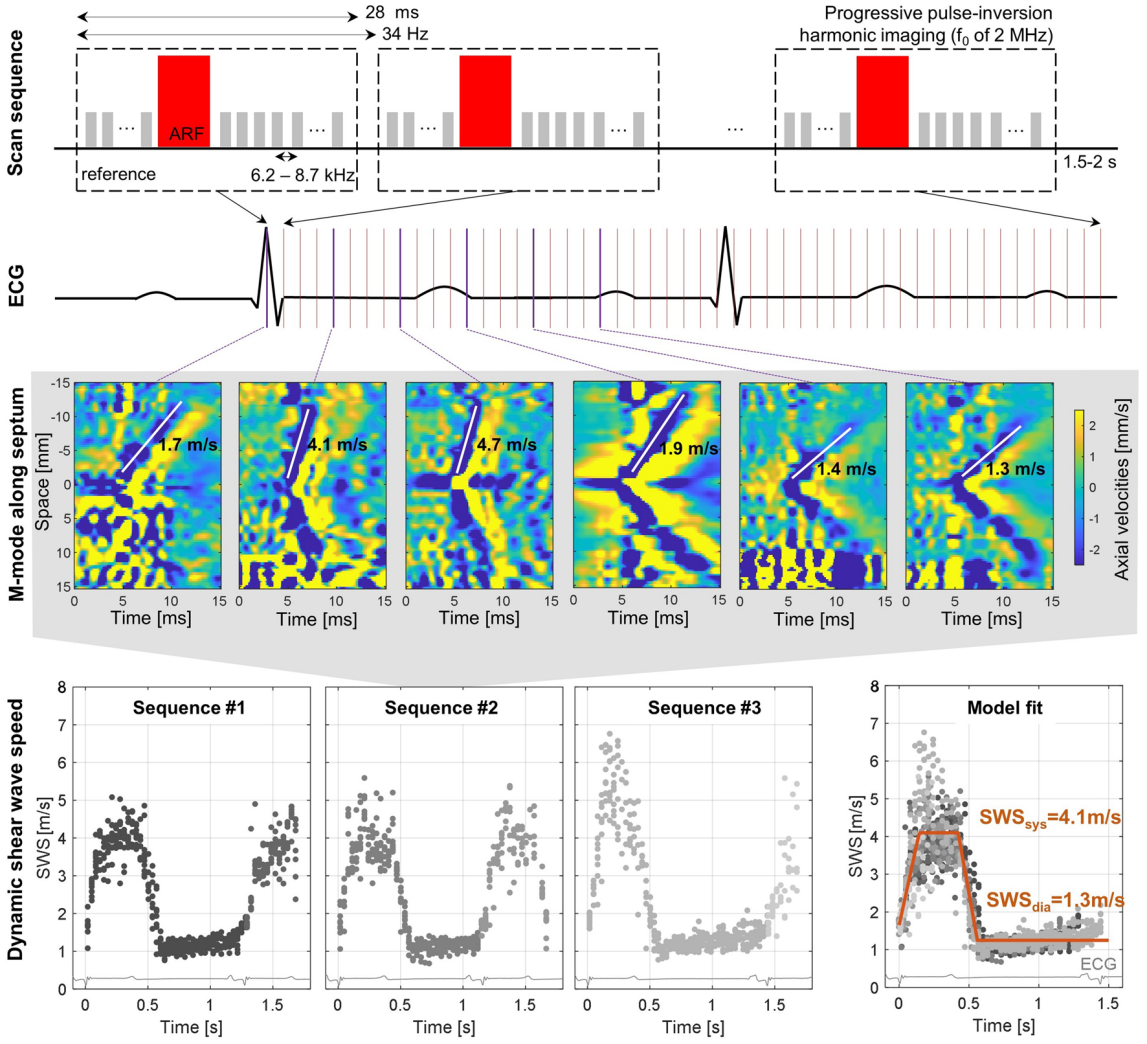
Shear wave elastography (SWE) sequence and postprocessing workflow. First row: schematic of a SWE imaging sequence, consisting of SWE acquisitions that were taken at 34 Hz during 1.5–2 s. Second row: schematic of an ECG signal. Third row: tissue velocity panels along the septum for different acquisitions at different time points, together with shear wave speed estimation. Fourth row: Shear wave speed data for three different SWE sequences at one intervention stage. The last panel demonstrates the procedure of obtaining diastolic and systolic shear wave propagation speed (SWS_dia_ and SWS_sys_) via piecewise linear model fitting. Different colors of grey represent different heartbeats. Spread of estimated wave speed at one time point represents variability across 10 anatomical M-lines drawn by the 2 observers.

Figure 2 legend,

“Shear wave elastography (SWE) sequence and postprocessing workflow. First row: schematic of a SWE imaging sequence, consisting of SWE acquisitions that were taken at 34 Hz during 1.5–2 s. Second row: schematic of an ECG signal. Third row: tissue velocity panels along the septum for different acquisitions at different time points, together with shear wave speed estimation. Fourth row: Shear wave speed data for three different SWE sequences at one intervention stage. The last panel demonstrates the procedure of obtaining diastolic and systolic shear wave propagation speed (SWS_dia_ and SWS_sys_) via piecewise linear model fitting. Different colors of grey represent different heartbeats. Spread of estimated wave speed at one time point represents variability across 10 anatomical M-lines drawn by the 2 observers.”

now reads:

“Shear wave elastography (SWE) sequence and postprocessing workflow. First row: schematic of a SWE imaging sequence, consisting of SWE acquisitions that were taken at 31 Hz during 1.7-2.2 s. Second row: schematic of an ECG signal. Third row: tissue velocity panels along the septum for different acquisitions at different time points, together with shear wave speed estimation. Fourth row: Shear wave speed data for three different SWE sequences at one intervention stage. The last panel demonstrates the procedure of obtaining diastolic and systolic shear wave propagation speed (SWSdia and SWSsys) via piecewise linear model fitting. Different colors of grey represent different heartbeats. Spread of estimated wave speed at one time point represents variability across 10 anatomical M-lines drawn by the 2 observers.”

In the Results section, under the subheading ‘Diastolic wave speed’,

“The diastolic wave speed for the different interventions is summarized in Fig. 5a, with a wave speed of 1.3 m/s in baseline. Significant changes in wave speed were only observed after ischemia injury (+ 57%), whereas other interventions did not significantly alter the wave speed (− 18% in preload decrease, + 5% in afterload increase, + 4% in preload increase and + 94% after reperfusion).”

now reads:

“The diastolic wave speed for the different interventions is summarized in Figure 5a, with a wave speed of 1.2 m/s in baseline. Significant changes in wave speed were only observed after ischemia injury (+57%), whereas other interventions did not significantly alter the wave speed (-17% in preload decrease, +5% in afterload increase, +4% in preload increase and +94% after reperfusion).”

“A similar correlation is found between SWS and operational chamber stiffness dP/dV (R = 0.57; *p* < 0.01 in Fig. 6b). SWE measurements during and after I/R injury—as depicted in orange in Fig. 6—showed a strong significant correlation to EDP (R = 0.68; *p* < 0.01), operational chamber stiffness dP/dV (R = 0.73; *p* < 0.01) and stiffness constant β (R = 0.50; *p* = 0.03). Diastolic wave speed is more sensitive to changes in intrinsic stiffness than in loading, as reflected by the larger slope of the regression line (0.8 vs. 0.35 in Fig. 6b).”

now reads:

“A similar correlation is found between SWS and operational chamber stiffness dP/dV (R=0.54; p<0.01 in Figure 6b). SWE measurements during and after I/R injury – as depicted in orange in Figure 6 – showed a strong significant correlation to EDP (R=0.68; p<0.01), operational chamber stiffness dP/dV (R=0.73; p<0.01) and stiffness constant β (R=0.50; p=0.036). Diastolic wave speed is more sensitive to changes in intrinsic stiffness than in loading, as reflected by the larger slope of the regression line (0.73 vs. 0.29 in Figure 6b).”

As the result, Figures 4, 5, 6 and 8 were incorrect. The original Figures 4, 5, 6 and 8 and accompanying legends appear below.

**Fig. 4:**
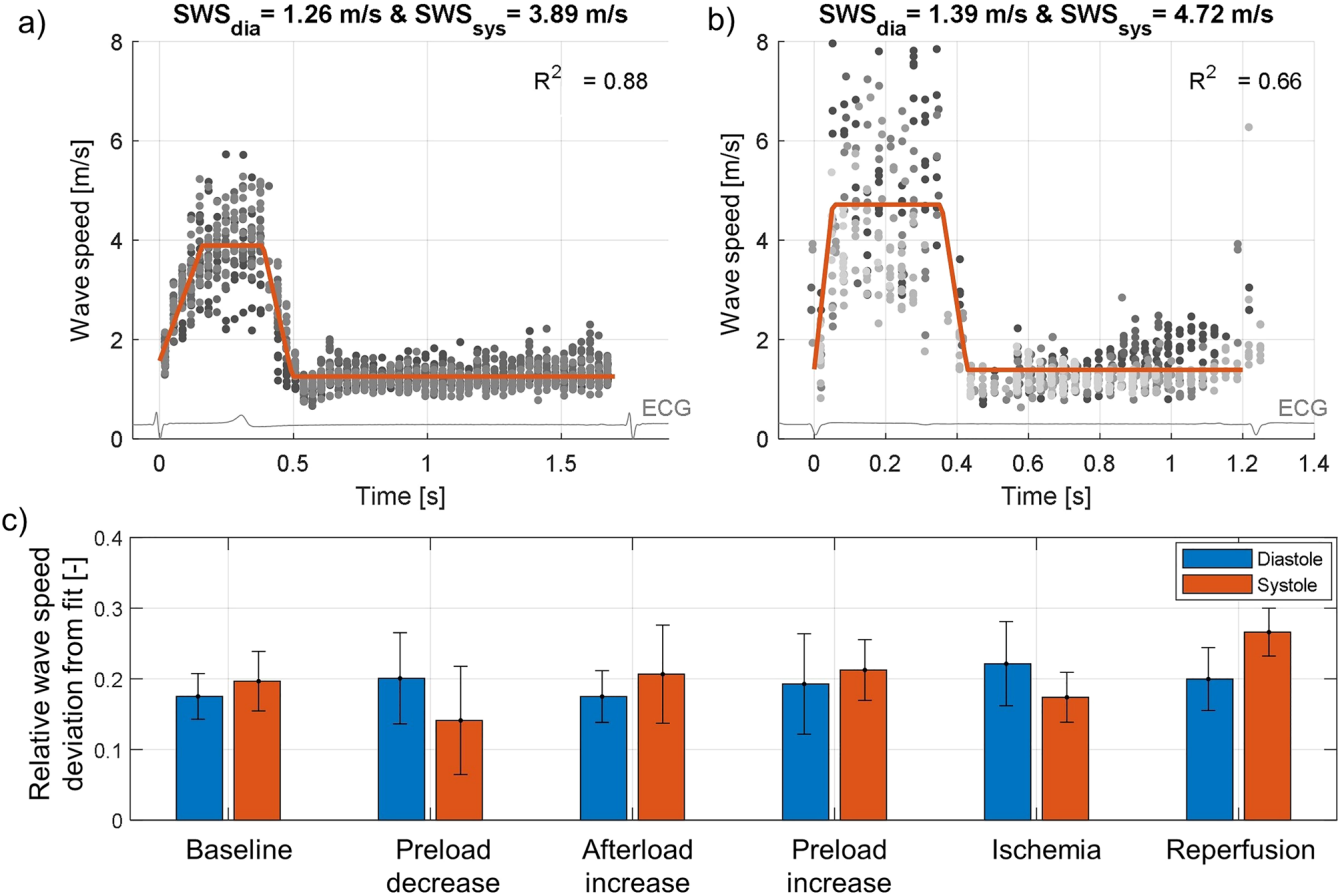
Variability of shear wave speed (SWS) estimation. (**a**) Example of low variability and good fit. (**b**) Example of high variability and moderate fit. (**c**) Averaged relative wave speed deviation from fit for all pigs is depicted for each condition and diastole/systole.

**Fig. 5:**
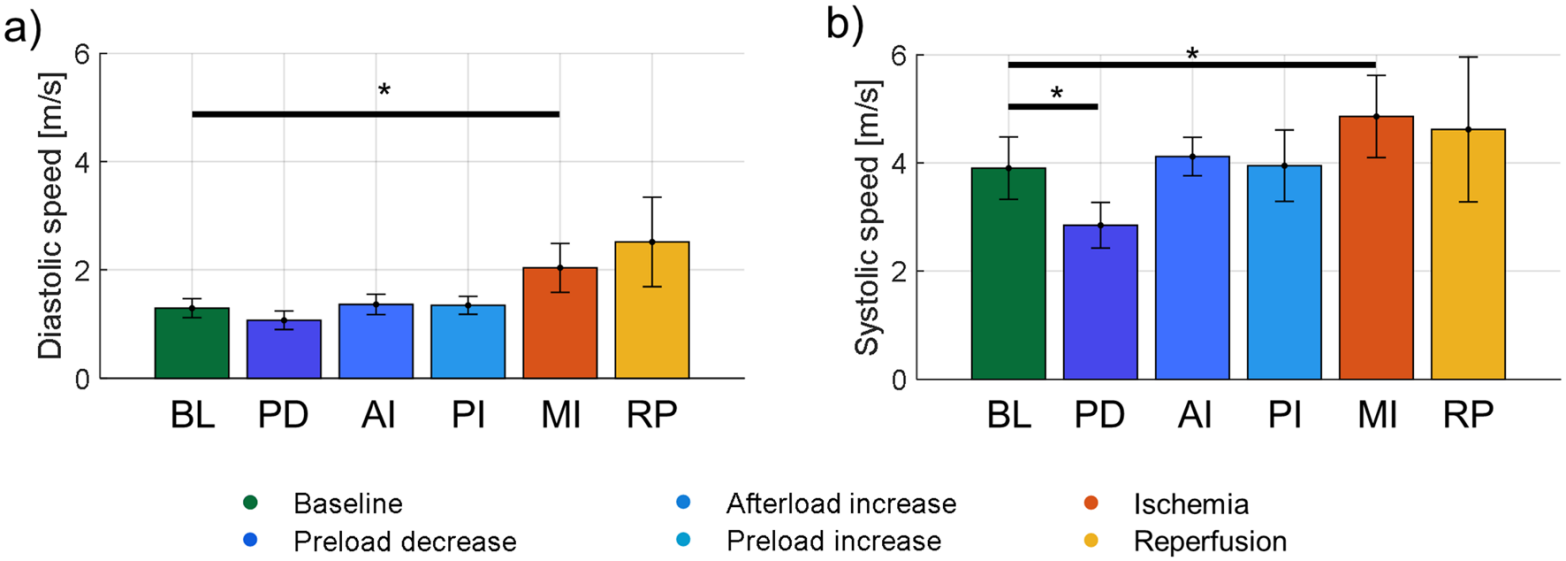
Diastolic and systolic wave speeds for the different interventions: baseline (BL), preload decrease (PD), afterload increase (AI), preload increase (PI), myocardial ischemia (MI) and reperfusion (RP). *p < 0.05 for t-test with Bonferroni correction.

**Fig. 6:**
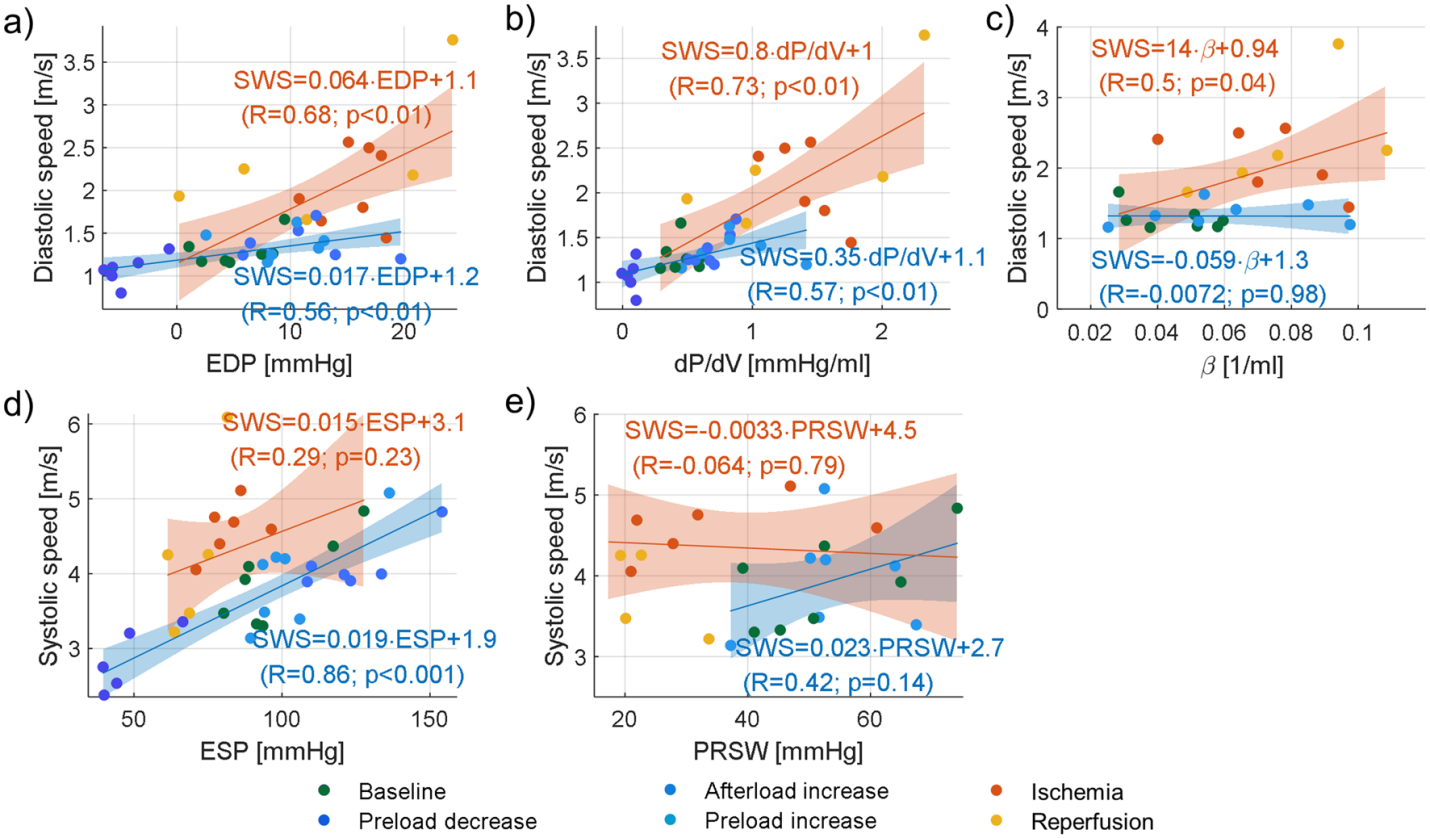
Correlations of diastolic and systolic wave speed, with (**a**) end-diastolic pressure (EDP), (**b**) operational stiffness (dP/dV), (**c**) stiffness constant β, (**d**) end-systolic pressure (ESP) and (**e**) preload-recruitable stroke work (PRSW) during loading (blue) and stiffness interventions (orange).

**Fig. 8:**
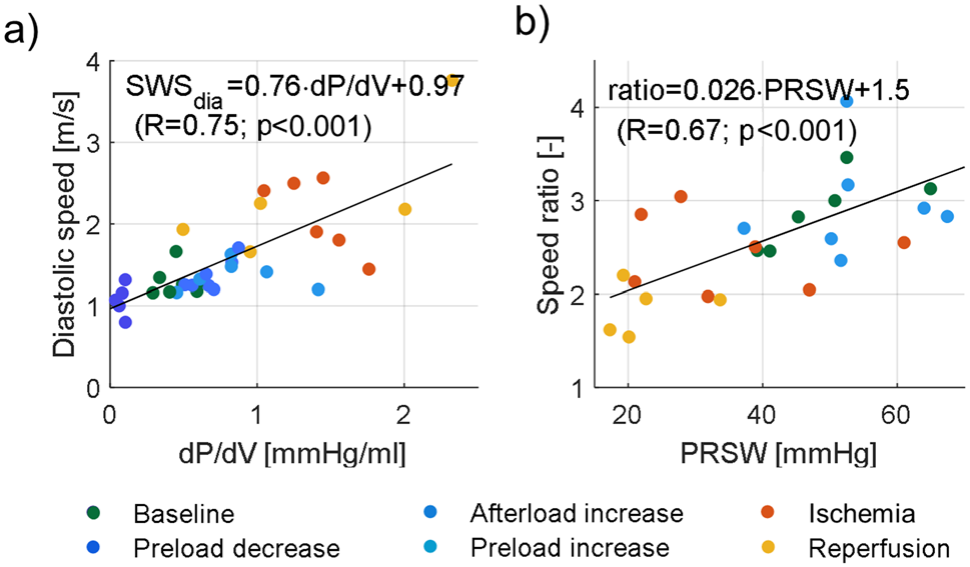
Correlation between diastolic wave speed and operational chamber stiffness (dP/dV) and between wave speed ratio and preload-recruitable stroke work (PRSW) for all interventions.

Additionally, Table 2 was incorrect. The original Table 2 and accompanying legends appear below.

**Table 2:** Linear regression results between diastolic wave speed (SWS_dia_) and end-diastolic pressure (EDP) on one hand and systolic wave speed (SWS_sys_) and end-systolic pressure (ESP) on the other hand during loading interventions, with goodness of fit R^2^.

Pig #	SWS_dia_ vs. EDP			SWS_sys_ vs. ESP		
	Slope (m/s/mmHg)	Intercept (m/s)	R^2^	Slope (m/s/mmHg)	Intercept (m/s)	R^2^
1	0.011	1.1	0.92	0.019	1.6	0.93
2	0.026	1.3	0.92	0.017	1.7	0.99
3	0.016	1.1	0.93	0.024	1.4	0.95
4	0.021	1.1	0.53	0.026	1.7	0.997
5	0.018	1.3	0.60	0.012	2.8	0.85
6	0.014	1.3	0.81	0.024	1.6	0.91
7	0.014	1.2	0.70	0.019	2.2	0.94
Mean	0.017 ± 0.005	1.2 ± 0.1	0.77 ± 0.17	0.020 ± 0.005	1.9 ± 0.5	0.94 ± 0.05

Under the subheading ‘Systolic wave speed’,

“Figure 5b shows the resulting systolic wave speed for all interventions, with a wave speed of 3.9 m/s at baseline.

The individual correlations with ESP are given in Table 2, and show in general a slightly higher goodness-of-fit (R^2^) than for the diastolic measurements, probably due to the limited sensitivity of the manual wave speed estimator to detect differences in speed (ΔSWS_dia_ = 0.37 m/s vs. ΔSWS_sys_ = 1.27 m/s).”

now reads:

“Figure 5b shows the resulting systolic wave speed for all interventions, with a wave speed of 3.5 m/s at baseline.

The individual correlations with ESP are given in Table 2, and show in general a slightly higher goodness-of-fit (R^2^) than for the diastolic measurements, probably due to the limited sensitivity of the manual wave speed estimator to detect differences in speed (ΔSWS_dia_ = 0.33 m/s vs. ΔSWS_sys_ = 1.14 m/s).”

In the Discussion section, under the subheading ‘Myocardial operational stiffness’,

“Our study confirms this earlier research: the change in the stiffness constant β of the EDPVR increased after the ischemia period (0.073 vs. 0.045 1/ml; *p* = 0.06) and increased even further after the reperfusion period (0.087 vs. 0.045 1/ml; *p* < 0.05), which was reflected in the change of diastolic wave speed after ischemia injury (2.0 vs. 1.3 m/s; *p* < 0.05) and reperfusion injury (2.5 m/s).

The difference in the slopes of the fitted linear regression curves in Fig. 6b (0.80 vs. 0.35) suggests that diastolic speed is more sensitive to changes in intrinsic characteristics than changes in loading.”

now reads:

“Our study confirms this earlier research: the change in the stiffness constant β of the EDPVR increased after the ischemia period (0.073 vs. 0.045 1/ml; p = 0.06) and increased even further after the reperfusion period (0.087 vs. 0.045 1/ml; p < 0.05), which was reflected in the change of diastolic wave speed after ischemia injury (1.8 vs. 1.2 m/s; p < 0.05) and reperfusion injury (2.3 m/s).

The difference in the slopes of the fitted linear regression curves in Figure 6b (0.73 vs. 0.29) suggests that diastolic speed is more sensitive to changes in intrinsic characteristics than changes in loading.”

Under the subheading ‘Contractility’,

“However, systolic wave speed increased significantly after ischemia injury (4.9 vs. 3.9 m/s; *p* = 0.01 in Fig. 6c), which does not correspond with the observed decline in contractility in terms of pressure–volume measures after the I/R injury (see Table 1).”

now reads:

“However, systolic wave speed increased significantly after ischemia injury (4.4 vs. 3.5 m/s; p=0.01 in Figure 5b), which does not correspond with the observed decline in contractility in terms of pressure-volume measures after the I/R injury (see Table 1).”

Further, in the original version of this Article Jürgen Duchenne was incorrectly affiliated with Affiliation 4. Their correct affiliation is Affiliation 5:

5. Cardiology, KU Leuven, Leuven, Belgium

Finally, the Funding section was incomplete,

“This work was supported by the Research Foundation Flanders (FWO, Brussels, Belgium) under Grant 1211620N to Annette Caenen and, Grants G092318N and 1832922N to Jens-Uwe Voigt. This work is also part of the TTW–Dutch Heart Foundation partnership program ‘Earlier recognition of cardiovascular diseases’ with project number 14740.”

now reads:

"This work was supported by the Research Foundation Flanders (FWO, Brussels, Belgium) under Grant 1211620N to Annette Caenen and, Grants G092318N and 1832922N to Jens-Uwe Voigt and Grant 12ZZN22N to Jürgen Duchenne. This work is also part of the TTW–Dutch Heart Foundation partnership program ‘Earlier recognition of cardiovascular diseases’ with project number 14740."

The original Article has been corrected.

