# Supplementary material for: Continuous shear wave measurements for dynamic cardiac stiffness evaluation in pigs
Source: Sci Rep. 2023 Oct 17;13:17660. doi: 10.1038/s41598-023-44588-4 (PMC10582168; doi:10.1038/s41598-023-44588-4)
Supplement: Supplementary file 1 — Supplementary Information. [file 41598_2023_44588_MOESM1_ESM.docx]

**Appendix: Fourier energy spectra**

To get a better understanding of the excited frequencies during wave propagation, a phase speed analysis was performed for several SWE acquisitions as shown in Figure 1. Therefore, the space-time plots were cropped to depict one shear wave propagation, and consequently the 2D Fast Fourier Transform was applied to obtain the Fourier spectra ^1^. The resulting spectra depict on average a dominant excited frequency of 146 Hz in diastole and 223 Hz in systole. The upper frequency bound, characterized by the frequency for which the energy has dropped 12 dB, is on average 317 Hz in diastole and 532 Hz in systole. This demonstrates a shift towards higher frequencies in systole. Additionally, the upper wavenumber bound, i.e. the wavenumber for which the energy has dropped 12 dB, is lower for systole than diastole (670 1/m vs. 1669 1/m), indicating a tilt of the energy band in the Fourier spectra. These results present initial findings, as the Fourier analysis was not consistently applied to all SWE data due to the limited signal-to-noise ratio.


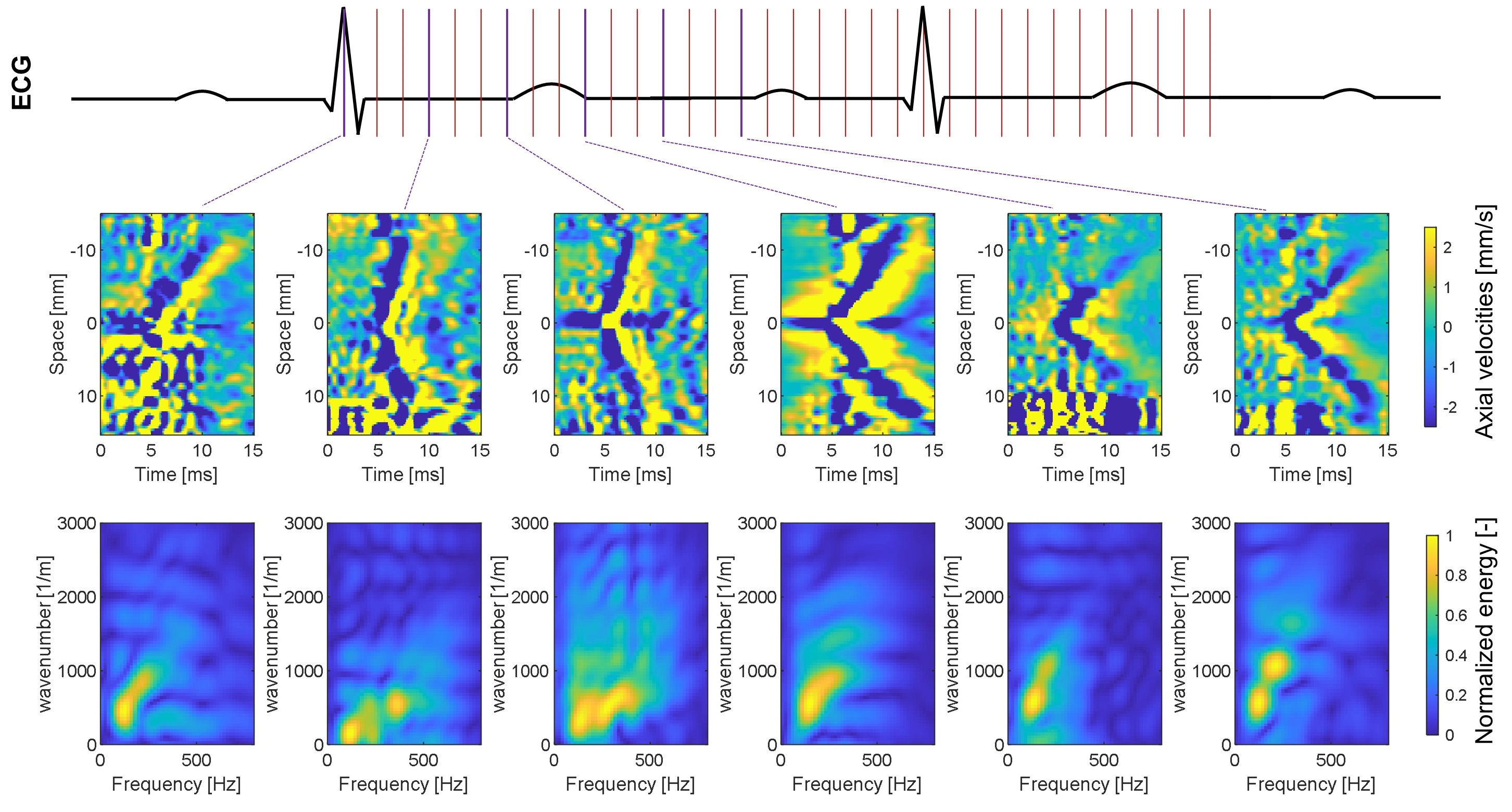


Figure 1 - Variations in Fourier energy across the cardiac cycle, in which the upper row of panels are taken from Figure 2. Only the upper shear wave, i.e. the wave traveling from base to apex, was considered for performing the Fourier analysis.

**References**

1 Bernal, M., Nenadic, I., Urban, M. W. & Greenleeaf, J. F. Material property estimation for tubes and arteries using ultrasound radiation force and analysis of propagating modes. *J Acoust Soc Am* **129**, 1344-1354, doi:10.1121/1.3533735] (2011).
